# Photodynamic Therapy Efficacy of the Human Papillomavirus-Related Cervical Lesions

**DOI:** 10.3390/jcm15041596

**Published:** 2026-02-19

**Authors:** Alexander Dushkin, Tatyana Grishacheva, Stanislav Afanasiev, Irina Dushkina, Alexander Karaulov, Elena Biryukova, Akmaral Khangeldi, Kristina Babayan, Nasrula Shanazarov, Maxim Afanasiev

**Affiliations:** 1 Department of General Medicine No. 1, Moscow Clinical Science and Research Center 52, 123182 Moscow, Russia; 2 Center of the Laser Medicine, First Pavlov State Medical University, 197022 Saint Petersburg, Russia; grishatanchik82@gmail.com; 3 Department of Medical Biotechnology, G.N. Gabrichevsky Moscow Research Institute for Epidemiology and Microbiology, Federal Service for the Oversight of Consumer Protection and Welfare, 125212 Moscow, Russia; afanasievss409.4@bk.ru; 4 Gynecology Department, Moscow City Hospital 67, 123423 Moscow, Russia; dush-ka@mail.ru; 5 Clinical Immunology and Allergology Department, I.M. Sechenov First Moscow State Medical University, 119991 Moscow, Russia; karaulov_a_v@staff.sechenov.ru (A.K.); rectorat@staff.sechenov.ru (E.B.); maxim.afanasyev78@gmail.com (M.A.); 6 Gynecology Department, NJSC “Astana Medical University”, Astana 010000, Kazakhstan; akhangeldi@mail.ru; 7 Medical Faculty, Rostov State Medical University, 344022 Rostov-on-Don, Russia; kristina20002000@list.ru; 8 Medical Center Hospital of President’s Affairs Administration of the Republic of Kazakhstan, Astana 010000, Kazakhstan; nasrulla@inbox.ru

**Keywords:** photodynamic therapy, HPV clearance, cervical lesions, lesion remission, complete response

## Abstract

**Background/Objectives**: This study aims to fill certain knowledge gaps by assessing the clinical effectiveness of PDT in a large group of women with HPV-related cervical lesions and examining how different patient factors affect treatment results. **Methods**: A total of 811 women aged from 19 to 76 were retrospectively analyzed who were treated by PDT of HPV infection with atypical squamous cells and HPV-related cervical lesions. PDT was performed using chlorin e6-based systemic photosensitizers. Irradiation was carried out at 662 nm. The endocervical dose was 334 J/cm^2^, and the ectocervical dose was 291 J/cm^2^. **Results**: Overall HPV clearance was 91.1%, lesion remission was 95.3%, and complete response was 88.3%, with the highest complete response observed in the HSIL group compared with HPV-positive ASCs. Multivariable models showed that multiple HPV infection (especially >3 genotypes) and pregnancy history were associated with lower odds of complete response, while younger age (18–25 years) and TZ2 were associated with higher odds of complete response. **Conclusions**: PDT using chlorin e6-based photosensitizers demonstrated high clinical and virological effectiveness across HPV-related cervical abnormalities, including HSIL, supporting its role as an organ-preserving treatment option. Multiple HPV genotypes and pregnancy history may identify patients at increased risk of partial response and warrant closer follow-up or tailored treatment strategies.

## 1. Introduction

Cervical cancer remains a major global health challenge arising from the malignant progression of human papillomavirus (HPV)-induced cervical intraepithelial neoplasia (CIN). Persistent high-risk HPV infection is the causal factor in virtually all cases of cervical dysplasia and cancer. Globally, cervical cancer ranks as the third most prevalent malignancy among women, with approximately 255,016 cases occurring in individuals aged 20 to 49 years. It is also the second leading cause of death from malignant diseases, accounting for 93,736 deaths [1]. The natural history of cervical intraepithelial neoplasia is heterogeneous. While the majority of CIN1 lesions regress spontaneously, the clinical course of high-grade lesions is less predictable. Recent systematic reviews indicate that approximately 30–40% of HSIL/CIN2+ lesions may persist or progress without treatment, with HSIL/CIN3 lesions showing particularly high persistence rates and a non-negligible long-term risk of invasive cancer development [2,3,4,5]. These data emphasize the importance of balancing oncological safety with the need to avoid overtreatment, especially in women of reproductive age. Standard therapies for CIN include excisional or ablative procedures [6], such as loop electrosurgical excision, cold-knife conization, cryotherapy and laser ablation, which have high success rates but can damage cervical structure [7,8,9]. Surgical treatments can lead to bleeding, infection, cervical stenosis, and major obstetric complications such as cervical insufficiency and preterm birth [3,10,11]. With the rising incidence of CIN in younger women and a global emphasis on fertility preservation, there is a clear need for non-invasive alternatives [12].

Photodynamic therapy (PDT) has emerged over the past decade as a promising non-surgical option for HPV-related cervical lesions. PDT involves the administration of a photosensitizer that selectively accumulates in dysplastic or HPV-infected epithelium, followed by illumination with a specific wavelength of light to produce reactive oxygen species that destroy abnormal cells while sparing healthy tissue [12,13]. Common photosensitizers, such as 5-aminolevulinic acid (5-ALA) and chlorin-based drugs, accumulate in neoplastic cells. When activated by red light (630–662 nm), they produce singlet oxygen and free radicals that trigger apoptosis and necrosis in dysplastic epithelium [14]. In addition to direct cytotoxicity, PDT may have immunomodulatory effects. Studies report increases in local T-cell activity and reduction in pro-inflammatory cytokines after PDT, suggesting enhanced clearance of HPV infection via immune mechanisms [15]. Unlike excisional methods, PDT treats lesions in situ without excising tissue, thus preserving the anatomic and functional integrity of the cervix [16]. This organ-sparing approach is particularly attractive for managing CIN in women who wish to avoid the reproductive risks of surgery.

This study aims to fill certain knowledge gaps by assessing the clinical effectiveness of PDT in a large group of women with HPV-related cervical lesions and examining how different patient factors affect treatment results.

## 2. Materials and Methods

### 2.1. Study Design and Ethical Considerations

**Figure 1:**
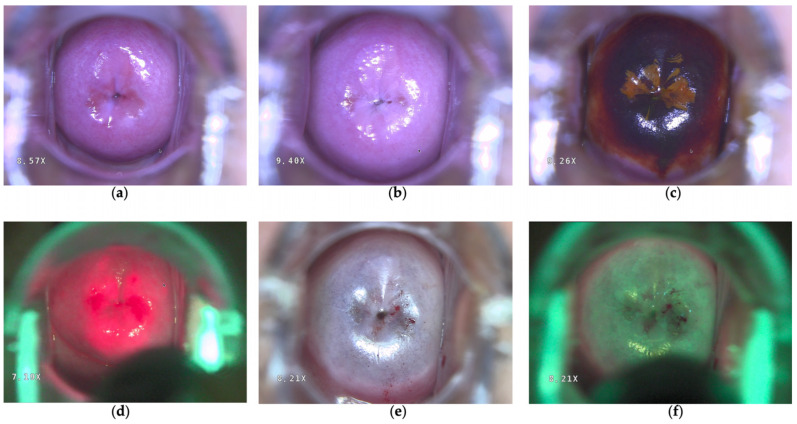
Colposcopy (**a**) with acetic acid (**b**) and Schiller’s test (**c**). Fluorescence diagnosis assisted by colposcopy (**d**) conducted before PDT, and photobleaching checked for control (**e**) after PDT. Cyanosis appeared on the cervical surface one minute after PDT (**f**).

A total of 811 electronic medical records of patients aged from 19 to 76 were retrospectively analyzed who were treated with one PDT session of HPV infection with atypical squamous cells (ASCs) and HPV-related cervical lesions from November 2016 to September 2024 in the gynecology department of Moscow privacy clinics. HPV infection was proven by HPV tests (HPV DNA using a validated PCR assay for high-risk HPV or the Digene test). HPV viral load was assessed using validated quantitative PCR assays and expressed in logarithmic values (log10 copies per sample), in accordance with the manufacturer’s recommendations. HPV testing was performed using validated and certified molecular diagnostic assays. Until June 2017, HPV detection was carried out using the cobas^®^ 4800 HPV test (Roche Diagnostics, Basel, Switzerland, FSR No. 2011/09492) in accordance with the instructions. Starting from June 2017, HPV testing was additionally performed using the test system manufactured by LLC VECTOR-BEST (Novosibirsk, Russia, FSR No. 2021/13457). This assay has an analytical sensitivity of 100 copies of HPV DNA. HPV-related cervical lesions were verified by liquid-based cytology (LBC), specifically the BD SurePath liquid-based Pap test (BD SurePath™, Franklin Lakes, NJ, USA, SKU/REF491452), and according to the Bethesda classification 2014. Patients with HSIL/CIN2+ results were directed to a precise incisional biopsy with a histological exam to exclude invasive cervical cancer. In cases of discordance between LBC and histology, patients were classified according to the higher-grade lesion identified by either diagnostic modality (worst-case approach). All patients underwent colposcopy examination (Figure 1a) with acetic acid (Figure 1b) and Schiller’s test (Figure 1c) before PDT. All patients included in the study were HIV-negative at the time of PDT.

Patients were divided into three groups based on morphological results (LBC and histological exam):

- A total of 75 women with HPV infection and ASCs: ASCs-US or ASCs-H;
- A total of 130 women with low-grade squamous intraepithelial lesions (LSILs);
- A total of 606 women with high-grade squamous intraepithelial lesions (HSILs).

### 2.2. PDT Protocol

PDT was performed after menstruation using chlorin e6-based photosensitizers registered in the Russian Federation: Photoditazine^®^ (LLC “Company “DEKO”, Plant Vyshny Volochyok, Russia, chlorin e6, Reg. No. LP-004885) or Fotolon^®^ (RUPE Belmedpreparaty, Minsk, Republic of Belarus, chlorin e6, Reg. No. P N015948/01). Drugs were administered intravenously 180–200 min before irradiation. Fluorescence diagnostics were performed with a 405 nm LED source and a yellow filter mounted on a colposcope (Kernel KN-2200A-HD, Kernel Medical Equipment Co., Ltd., Xuzhou, China) to visualize photosensitizer accumulation (Figure 1d). Irradiation was carried out with the LAKHTA-MILON laser system (MILON Group, Moscow, Russia) at 662 nm. The endocervical dose was 334 J/cm^2^ (cylindrical diffuser fiber), and the ectocervical dose was 291 J/cm^2^ (microlens applicator). Post-procedure fluorescence confirmed photosensitizer photobleaching (Figure 1e,f). PDT was performed under local anesthesia. In addition, spinal anesthesia was administered upon patient request to ensure greater comfort during the procedure, particularly in patients with increased pain sensitivity or anxiety. All patients adhered to a 48 h dark regimen, including avoidance of bright light sources and use of sunscreen when outdoors.

### 2.3. Follow-Up and Outcome Assessment

**Figure 2:**
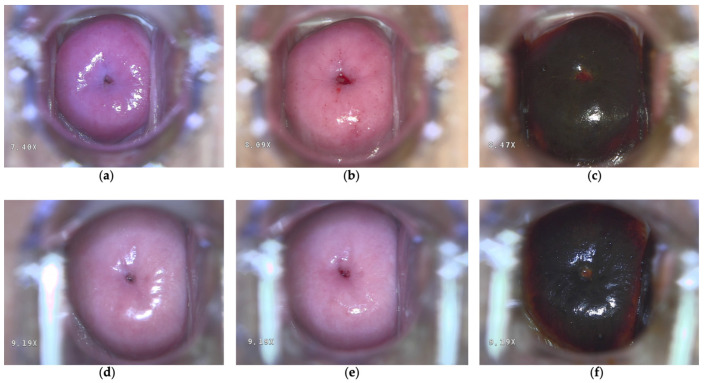
Colposcopy examination (**a**,**d**) utilizing acetic acid application (**b**,**e**) and Schiller’s test (**c**,**f**) was performed at two months (**a**–**c**) and twenty-four months (**d**–**f**) following photodynamic therapy (PDT).

The clinical efficacy of PDT was assessed using HPV testing and liquid-based cytology at the 3rd, 6th, and 12th months, and then annually thereafter. A complete response (CR) was defined as the simultaneous fulfillment of both criteria: (1) a negative high-risk HPV test (no detectable HPV DNA by validated PCR assay or the Digene test) and (2) negative cytology for intraepithelial lesions or malignancies (NILM) according to the Bethesda classification. HPV clearance and lesion remission were determined as the absence of persistent HPV and NILM, respectively. CR, HPV clearance and lesion remission were assessed at the patient’s last follow-up visit (from 1 to 103 months). Colposcopic examination with acetic acid application and Schiller’s test were conducted at each follow-up visit (Figure 2a–f).

### 2.4. Statistical Analysis

Data collection was performed in software Microsoft 365 v.2.106.3 (package Excel). Statistical analysis was performed using the programming language Python v.3.13 and the interactive development environment Visual Studio Code v.1.102. Quantitative variables were assessed for normality using the Shapiro–Wilk test. Quantitative variables were described using the mean (M) with standard deviation (SD) and the median (Me) with interquartile range (IQR). Categorical data were described with absolute and relative frequencies. Comparisons of three or more groups on a quantitative variable whose distribution differed from normal were made using the Kruskal–Wallis test and Dunn’s test with Holm correction as a post hoc method. Comparison of frequencies in the analysis of n by m contingency tables was performed using Pearson’s chi-square test. Post hoc comparisons were conducted using the Pearson chi-square test with Holm’s correction. The development of a prognostic model for the probability of a binary outcome was carried out using logistic regression. Nagelkerke pseudo-R^2^ was used as a measure of the model performance. To assess the discriminative ability of quantitative variables in predicting a binary outcome, the ROC curve analysis method was used. The cutoff value of the quantitative variable was determined by the highest value of the Youden index. The estimation of the patient survival function was conducted using the Kaplan–Meier method. Survival analysis of patients was conducted using Cox regression, which involves predicting the instantaneous risk of an event occurring at a specific point in time (hazard). Hazard ratios (HR) with 95% confidence intervals (95% CI) were calculated, and the statistical significance of the association with predictors was evaluated. The “event” was defined as an HPV positive/SIL+ or both. Patients without an event by the end of the study or loss to follow-up were censored at their last follow-up date. Differences were considered statistically significant at *p* < 0.05.

## 3. Results

### 3.1. Baseline Characteristics

**Figure 3:**
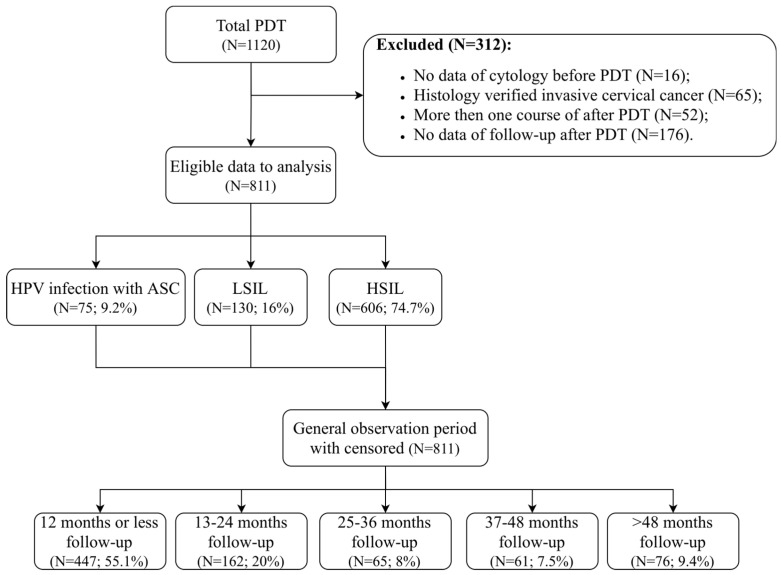
The trial flowchart of the study subjects. ASCs—atypical squamous cells; HPV—human papillomavirus; HSIL—high-grade squamous intraepithelial lesion; LSIL—low-grade squamous intraepithelial lesion.

The 811 patients were treated by PDT and divided into the HPV infection with ASCs (*n* = 75; 9.2%), LSILs (*n* = 130; 16%) and HSILs (*n* = 606; 74.7%) groups. Patients with HSILs had CIN2 in 24.4% (*n* = 198) of cases and CIN3 in 50.3% (*n* = 408). The trial flowchart is presented in Figure 3. The median follow-up period was 9.6 months (IQR: 3.3–23.4; min-max: from 1 to 103). Univariate analysis of baseline characteristics (demographic data, HPV status and viral load, LBC/histology results) is shown in Table 1.

Across the three study groups, several clinically relevant differences were observed in demographic variables, reproductive history and HPV characteristics, some of which were statistically significant.

Patients with HSILs were significantly older than participants with HPV and ASCs (34.9 ± 8.2 vs. 31.4 ± 7.2, *p* < 0.001) and LSILs (34.9 ± 8.2 vs. 31.4 ± 8 years, *p* < 0.001). A similar pattern was seen in the age-stratified analysis: the HSIL group had a higher proportion of participants aged 36–45 and ≥46 years compared with patients with HPV infection with ASCs and LSILs (*p* < 0.001).

The age at sexual debut differed slightly across groups, with significance detected only for mean values (*p* = 0.047). When we categorized age at sexual debut <18 vs. ≥18 years, no statistically significant differences were observed (*p* = 0.24). Both in mean and median values, the number of sexual partners was higher in the HSIL group (*p* = 0.011), with a particularly notable difference when compared with Group I (3 [1–5] vs. 4 [2–7], *p* = 0.037). The categorical distribution of sexual partners (1, 2–3, and >3) was not significantly different (*p* = 0.13).

Pregnancy status varied significantly across the groups. A higher proportion of women in Group III reported a history of pregnancy compared with Groups I (72.9% vs. 58.3%, *p* = 0.019) and II (72.9% vs. 57.6%, *p* = 0.002). The number of pregnancies also differed between groups (*p* < 0.001). However, categorized gravidity (1, 2–3, >3 pregnancies) did not show statistically significant group differences (*p* = 0.15).

The distribution of transformation zone (TZ) types (TZ1, TZ2, and TZ3) was similar across all three groups, with no significant differences detected (*p* = 0.84).

*HPV16* was the predominant genotype in all groups, with the highest prevalence in Group III compared with Group I (67.7% vs. 50%, *p* = 0.017) and Group II (67.7% vs. 47.9%, *p* < 0.001). *HPV18*, *HPV31*, and *HPV33* were detected less frequently and show comparable distributions. Patients with HSILs (Group III) had the highest proportion of single infections (51.5%), whereas Group II exhibited the highest proportion of single infections (41.5%). HPV-negative results were most common in Groups I and II (18.7 and 10.8%) and the least frequent in Group III (5.6%). Among individuals with multiple infections, the number of concurrent HPV types (2–3 vs. >3) did not differ significantly between groups (*p* = 0.86).

Although mean viral load values showed considerable variability and were not significantly different (*p* = 0.59), the median viral load was higher in Group II and Group III than in Group I (6.1 [5.4–7.1] and 6 [5.1–7] vs. 5.4 [4.5–6.1], *p* = 0.043 for both comparisons).

### 3.2. Comparison of Complete Response, HPV Clearance and Lesion Remission

**Figure 4:**
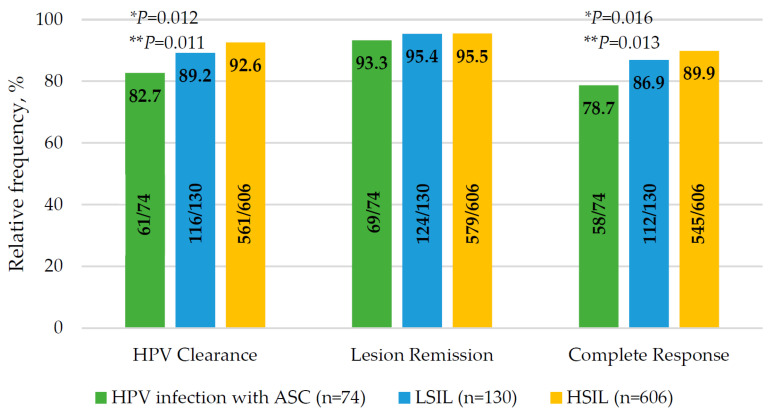
Overall response rate of HPV elimination, cytological outcome and complete response. ASCs—atypical squamous cells; HPV—human papillomavirus; HSIL—high-grade squamous intraepithelial lesion; LSIL—low-grade squamous intraepithelial lesion. * Difference between three groups; ** difference between HSILs and HPV infection with ASC groups.

The HPV clearance was 91.1% (*n* = 739). The lowest rate of HPV clearance was in the group of HPV infection with ASCs (*p* = 0.012, Figure 4). In pairwise comparison, HPV infection with ASCs and HSILs had a statistically significant difference (82.7% vs. 92.6%; *p* = 0.011).

The lesion efficacy was 95.3% (*n* = 773). The equal lesion efficacy was for each group (*p* = 0.68, Figure 4). Regression of cervical lesions was in 3.8% for HSILs and 0.8% for LSILs. Ineffective PDT was in 1.3% for HPV infection with ASCs, 3.8% for LSIL, and 0.7% for HSILs. Progression of cervical lesions was detected in 5.3% of cases for HPV infection with ASCs.

The complete response was 88.3% (*n* = 716). The highest rate of complete response was in the HSIL group (*n* = 544; 89.8%) compared with the LSIL group (*n* = 113; 86.9%) and HPV infection with ASCs (*n* = 59; 78.7%). In pairwise comparison, complete response rate was statistically higher in the HSIL group than in the HPV infection with the ASCs group (*p* = 0.011, Figure 4).

### 3.3. Multivariate Analysis of Factors for Complete Response, HPV Clearance and Lesion Remission

Multivariate analysis of factors for complete response (CR), HPV clearance and lesion remission was performed using logistic regression with stepwise factor selection (Table 2). Input predictors were age groups, age at sexual debut groups, number of sexual partners, pregnancy status, TZ types, HPV infection and grade of squamous intraepithelial lesion.

#### 3.3.1. Complete Response

**Figure 5:**
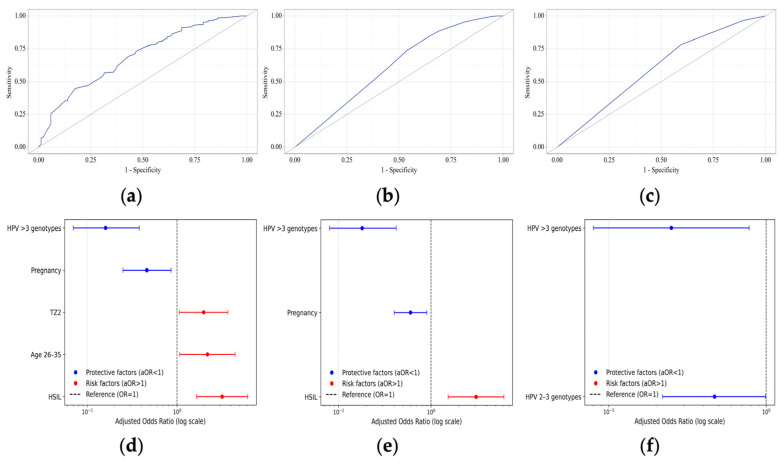
ROC curves of (**a**) complete response, (**b**) HPV clearance and (**c**) lesion remission and adjusted odds ratio for (**d**) complete response, (**e**) HPV clearance and (**f**) lesion remission.

After adjustment for covariates, several factors were independently associated with the odds of CR (Table 3, Figure 5d). Patients aged 18–25 years had significantly higher odds of achieving CR compared with the other age groups (OR = 2.18; 95% CI: 1.07–4.44; *p* = 0.032). A TZ2 was also associated with increased odds of CR relative to TZ1 (OR = 1.98; 95% CI: 1.06–3.69; *p* = 0.033), whereas TZ3 showed no significant association. In contrast, pregnancy status was independently associated with a reduced likelihood of CR (OR = 0.46; 95% CI: 0.25–0.86; *p* = 0.014). The number of HPV genotypes exhibited a strong inverse dose-dependent association with CR. Compared with single HPV infection, infection with two to three HPV genotypes was associated with a significant reduction in CR odds (OR = 0.44; 95% CI: 0.25–0.77; *p* = 0.004) while infection with more than three HPV genotypes showed a more pronounced decrease (OR = 0.16; 95% CI: 0.07–0.38; *p* < 0.001). Baseline cytological diagnosis was also significantly associated with treatment outcomes. Compared with HPV-positive cases with ASCs, both LSILs (OR = 2.47; 95% CI: 1.10–5.54; *p* = 0.029) and HSILs (OR = 3.18; 95% CI: 1.65–6.12; *p* = 0.001) were associated with increased odds of CR.

ROC analysis demonstrated moderate discriminative performance with an AUC of 0.69 (95% CI: 0.64–0.74; *p* < 0.001, Figure 5a). At the optimal cutoff probability (*p* = 0.89, Youden index), the model of logistic regression achieved a sensitivity of 61% and specificity of 62.8%.

#### 3.3.2. HPV Clearance

After adjustment, infection with more than three HPV genotypes was strongly associated with reduced odds of HPV clearance (OR = 0.18; 95% CI: 0.08–0.42; *p* < 0.001, Figure 5e). Infection with two to three HPV genotypes was not significantly associated with HPV clearance. Baseline cytology diagnosis demonstrated an independent association with outcome. Compared with HPV-positive cases with ASCs, HSILs were associated with significantly higher odds of HPV clearance (OR = 3.07; 95% CI: 1.53–6.13; *p* = 0.002, Figure 5e), while LSIL results did not reach statistical significance.

ROC analysis showed limited but statistically significant discrimination with an AUC of 0.62 (95% CI: 0.55–0.67; *p* < 0.001, Figure 5b). At the optimal cutoff probability (*p* = 0.94, Youden index), the model of logistic regression achieved a sensitivity of 60.3% and specificity of 55.4% (Table 4).

#### 3.3.3. Lesion Remission

Lesion remission was associated with multiple HPV infections compared with a single HPV infection (Table 5, Figure 5f). Infections with more than three HPV genotypes (AOR = 0.25; 95% CI: 0.08–0.78; *p* = 0.017) and two to three HPV genotypes (AOR = 0.47; 95% CI: 0.22–0.99; *p* = 0.047) showed a strong negative association in the odds of lesion remission.

ROC analysis showed low but statistically significant discrimination with an AUC of 0.60 (95% CI: 0.51–0.69; *p* = 0.006, Figure 5c). At the optimal cutoff probability (*p* = 0.96, Youden index), the model of logistic regression achieved a sensitivity of 78.2% and specificity of 40.5%.

### 3.4. Time-to-Event for Partial Response, Persistent HPV Infection and Persistent Lesions

Time-to-event analyses were performed to evaluate predictors of partial response, persistent HPV infection, and persistent lesion following PDT. Kaplan–Meier survival curves were compared using likelihood ratio testing and multivariable Cox proportional hazards models were applied to identify independent predictors. Input predictors were age groups, age at sexual debut groups, number of sexual partners, pregnancy status, TZ types, HPV infection and grade of SILs.

#### 3.4.1. Partial Response

**Figure 6:**
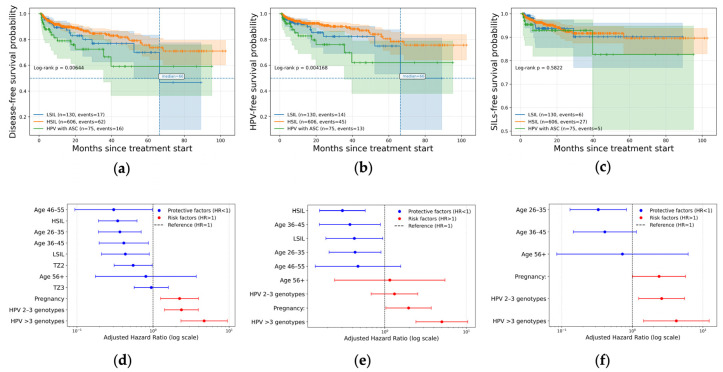
Kaplan–Meier curves of (**a**) disease-free, (**b**) HPV-free, and (**c**) SILs-free survival probabilities depending on groups and adjusted hazard ratio for (**d**) complete response, (**e**) HPV clearance, and (**f**) lesion remission.

For partial response, Kaplan–Meier analysis (Figure 6a) revealed significant differences in disease-free survival across age groups, pregnancy status, number of HPV genotypes, TZ types and baseline cytology (*p* < 0.001). The median time to partial response was reached only in patients aged 18–25 years, with a median of 66.4 months, whereas the median was not reached in patients aged 26–35, 36–45, and 46–55 years. In patients aged ≥56 years, the median was also not reached. According to the transformation zone type, the median time to partial response was not reached in all TZ groups; however, earlier declines in response-free survival were observed in TZ1 compared with TZ2. Pregnant patients demonstrated an earlier occurrence of partial response. In this subgroup, the median time to partial response was 61.0 months, whereas the median was not reached in non-pregnant patients. A pronounced effect was observed for HPV multiplicity. Patients infected with more than three HPV genotypes reached a median time to partial response of 61.0 months, while the median was not reached in patients with single-genotype or two-to-three-genotype infections. Baseline cytology was also associated with time-to-event outcomes. The median time to partial response was not reached in patients with LSILs and HSILs, whereas earlier events were observed in patients with HPV-positive atypical squamous cells.

In multivariable Cox regression (Figure 6d and Table 6), the ages of 26–35 years (HR = 0.37; 95% CI: 0.19–0.70), 36–45 years (HR = 0.41; 95% CI: 0.20–0.87), and 46–55 years (HR = 0.30; 95% CI: 0.09–0.98) were independently associated with a reduced risk of partial response. TZ2 was associated with lower hazard compared with TZ1 (HR = 0.55; 95% CI: 0.31–0.97). Pregnancy increased the risk of partial response (HR = 2.23; 95% CI: 1.26–3.95). Infection with two to three HPV genotypes (HR = 2.36; 95% CI: 1.42–3.93) and more than three genotypes (HR = 4.70; 95% CI: 2.33–9.48) significantly increased risk in a dose-dependent manner. Baseline LSILs and HSILs were associated with reduced hazards compared with HPV-positive ASCs.

#### 3.4.2. Persistent HPV Infection

For persistent HPV infection, Kaplan–Meier curves (Figure 6b) demonstrated significant stratification by age, pregnancy status, HPV multiplicity, and baseline diagnosis (*p* < 0.001). Patients aged 18–25 years demonstrated earlier HPV persistence, whereas the median HPV-free survival was not reached in patients aged 26–35 and 36–45 years. In older age groups, median estimates were also not reached. Pregnant patients exhibited earlier HPV persistence, with a median HPV-free survival of approximately 58.7 months, while the median was not reached in non-pregnant patients. HPV multiplicity had a strong impact on median survival. Patients with more than three HPV genotypes reached a median time to HPV persistence of 49.8 months, whereas the median was not reached in patients with single-genotype or two-to-three-genotype infections. By baseline cytology, the median HPV-free survival was not reached in patients with LSILs and HSILs, while earlier HPV persistence was observed in patients with HPV-positive ASCs.

In Cox regression (Figure 6e and Table 7), age 26–35 years (HR = 0.43; 95% CI: 0.21–0.90) and 36–45 years (HR = 0.37; 95% CI: 0.16–0.89) were associated with reduced risk of persistent HPV infection. Pregnancy increased the hazard of HPV persistence (HR = 1.96; 95% CI: 1.03–3.72). Infection with more than three HPV genotypes was the strongest predictor of HPV persistence (HR = 5.01; 95% CI: 2.41–10.42). Baseline LSILs and HSILs were associated with significantly reduced hazards of HPV persistence.

#### 3.4.3. Persistent Lesions

Kaplan–Meier (Figure 6c) analysis showed significant differences in lesion-free survival depending on various factors: multiple HPV infection, pregnancy status and age group. Patients with more than three HPV genotypes reached a median time to lesion persistence of 52.3 months, whereas the median was not reached in patients with single-genotype infection. Pregnancy status was associated with earlier lesion persistence. In pregnant patients, the median lesion-free survival was approximately 54.1 months, while the median was not reached in non-pregnant patients. Age-stratified analysis showed that patients aged 26–35 years did not reach the median lesion-free survival during follow-up, whereas earlier events were observed in younger patients.

In multivariable Cox regression (Figure 6f and Table 8), infection with two to three HPV genotypes (HR = 2.61; 95% CI: 1.23–5.52) and more than three genotypes (HR = 4.21; 95% CI: 1.44–12.31) independently increased the risk of persistent SIL. Pregnancy was associated with increased SIL persistence (HR = 2.40; 95% CI: 1.01–5.70), while age 26–35 years was associated with reduced risk (HR = 0.33; 95% CI: 0.13–0.83).

PDT was generally well tolerated. Cervical canal stenosis (atresia) developed in 6 patients (0.74%) as a late local adverse event. In all affected cases, cervical dilatation was performed approximately 2 months after PDT with satisfactory clinical outcomes.

## 4. Discussion

In this study, we evaluated PDT as a treatment for HPV-related cervical lesions: HPV infection with ASCs, LSILs and HSILs. The results demonstrate that PDT is highly effective in achieving both HPV clearance and lesion remission, corroborating and extending findings from prior research. We observed an overall CR rate of 88.3% in our cohort (716/811 patients), defined stringently as concurrent clearance of high-risk HPV and regression to normal cytology. This included HPV clearance in 91.1% of patients and lesion remission in 95.3%. Notably, the therapeutic efficacy was high even for patients with high-grade disease: among women with HSILs (CIN2+), PDT achieved a CR in 89.8% of cases, which is remarkably consistent with clearance rates reported for surgical excision in this population [16,17]. Our finding that the HSIL group had the highest response proportion (89.8% CR) aligns with several studies indicating that PDT can successfully eradicate severe dysplasia. For example, Hillemanns et al. reported a 95% response in CIN2 with hexaminolevulinate-PDT in a phase II trial [18], and more recent comparative studies have shown 82–100% remission of CIN2/3 lesions with ALA-PDT, which is comparable to conization [16]. Importantly, none of the HSIL cases in our series showed progression to invasive cancer during follow-up, reinforcing that PDT, when effective, halts disease progression in high-grade lesions.

Patients with LSILs and those with only atypical squamous cells (ASCs-US/ASCs-H with positive HPV) also benefited substantially from PDT in our study, though their CR rates were somewhat lower (87% for LSILs and 79% for HPV positive with ASCs). The slightly reduced success in the mild abnormality groups may reflect the challenge in treating low-grade infections that might be multifocal or the fact that some low-grade changes can persist due to reinfection. It is worth noting, however, that the lesion remission rate in LSILs was very high (99.2% showed no residual LSIL cytology after PDT), with only <1% showing any lesion persistence or progression. Our LSIL outcomes are consistent with other reports of proactive treatment in CIN1. Li et al. observed a 94.8% pathological regression in LSILs at 6 months post-ALA PDT [13], and a Brazilian trial of MAL-PDT noted 75% of CIN1 cleared at 2 years (versus 57% in a placebo group) [19]. Historically, some randomized studies found no advantage of PDT over observation in CIN1 (owing to high spontaneous regression) [18]. Our data, however, along with recent cohorts from China, suggest that for patients who desire active treatment of persistent LSILs or concomitant HR-HPV infection, PDT can effectively eliminate low-grade lesions and the virus. This proactive approach may alleviate patient anxiety and potentially reduce the risk of those 10–20% of LSILs that would otherwise progress [13].

One of the strengths of our study is the investigation of clinical and demographic factors that might influence PDT success. Multivariable analysis identified several independent predictors of treatment outcome, and these findings generally concur with trends noted in the literature. Perhaps the most significant factor was HPV infection multiplicity. Patients harboring multiple high-risk HPV genotypes had substantially lower odds of CR and higher hazard of treatment failure over time, in a dose-dependent fashion (adjusted OR 0.16 for >3 types vs. single-type HPV infection). Similarly, in our time-to-event analysis, infection with ≥2 HPV strains markedly increased the risk of an “partial response” event (adjusted HR 2.36 for 2–3 types and 4.70 for >3 types). These results reinforce a consensus that co-infection with multiple HPV strains is a key risk factor for therapeutic failure. Multiple HPV infections have been shown in other studies to predict lower clearance rates and higher recurrence after treatment [16]. For instance, Wang et al. noted that having more than one HPV strain was associated with suboptimal 6-month outcomes after PDT (OR < 0.1 for cure) [16]. In the context of genital warts (another HPV disease treated with PDT), patients with mixed low- and high-risk HPV required prolonged therapy and had lower virological clearance (50% clearance for high-risk vs. ~77% for low-risk HPV) [20], again indicating that high-risk/multiple HPV infections are more resilient [20]. Biologically, multiple infections could signify a heavier viral load or more compromised local immunity, thus making eradication by a single modality more challenging. Clinically, our findings highlight that patients with multiple HPV genotypes may need closer follow-up and possibly adjunctive measures (such as immunotherapy or repeat PDT sessions) to achieve complete clearance [16]. Other known cofactors of HPV persistence, such as smoking and immunosuppression, could not be evaluated in the present study and should be addressed in future prospective analyses.

Another factor affecting PDT outcomes was the cervical TZ type, which relates to lesion location and accessibility. We found that lesions with a TZ2 (partially endocervical, but still visible) responded better to PDT than those with a TZ1 (fully exocervical). TZ2 was associated with higher odds of CR (OR = 1.98) and a significantly lower hazard of recurrence (HR 0.55 vs. TZ1). Lesions involving the endocervical canal more deeply (TZ3) showed a trend toward lower response as well, though in our logistic model, TZ3 was not statistically different from TZ1. These observations concur with clinical experience that endocervical extension can hinder treatment efficacy. A recent study by Qian et al. explicitly identified cervical canal involvement as an independent risk factor for persistent HPV after ALA-PDT for HSILs [12]. In their cohort, patients whose HSILs extended into the endocervix had significantly higher rates of HPV persistence at 3 months (i.e., reduced clearance) [12]. Consistently, a specialized analysis of PDT for HSILs confined to the endocervical canal noted that while PDT can still be effective, careful delivery (e.g., intracervical fiber optic irradiation) is required [17]. The clinical implication is that women with TZ3 or largely endocervical lesions might benefit from modified PDT techniques (such as using an intrauterine light applicator or increasing photosensitizer incubation time) to ensure adequate treatment of the transformation zone. It may also be prudent to monitor such patients more rigorously post-PDT, as our survival curves suggested earlier loss of response in TZ3 lesions compared to more ectocervical ones.

Patient age and reproductive history also emerged as relevant factors, though their influence is nuanced. In our logistic regression, younger age (18–25 years) was associated with higher odds of initial CR, possibly reflecting a robust immune response or regenerative capacity in younger patients, and, indeed, other authors have noted that ALA-PDT is “particularly suitable for young women” with CIN because it spares fertility and appears effective in this group [12]. Paradoxically, however, our longitudinal analysis found that the youngest patients had the shortest disease-free intervals: women under 25 experienced incomplete response events sooner than older age groups (median 66.4 months for age 18–25 vs. not reached for older groups). Cox modeling confirmed that patients aged 26–45 had significantly lower hazards of recurrence than those under 25. One interpretation of this discrepancy is that while young patients respond well initially to PDT, they may be at higher risk of reinfection or relapse due to behavioral factors (more sexual exposure over time) or a cervical epithelium more prone to new HPV infection. In contrast, women over 30, once cleared, might have factors that make re-infection less frequent. Previous studies have not consistently noted age as a determinant of PDT outcome, so our findings provide novel insight and suggest counselling younger patients on diligent HPV prevention post-therapy.

Pregnancy status was associated in our study with a lower likelihood of complete response to PDT (adjusted OR = 0.46) and a higher risk of recurrence (HR = 2.23). An additional and clinically plausible explanation relates to cervical anatomy in parous women. Pregnancy and vaginal delivery may be associated with a broader or everted transformation zone and delivery-related lacerations or surface irregularities of the cervix, potentially affecting lesion topography and accessibility [21]. Cervical ectropion, a common finding in reproductive-age women influenced by hormonal status, may further modify the distribution of columnar and metaplastic epithelium at the ectocervix [22]. Because the cytotoxic effect of PDT is spatially restricted to photosensitizer-enriched and adequately illuminated tissue, non-uniform photosensitizer coverage or light delivery over an irregular cervical surface could theoretically reduce treatment uniformity and contribute to HPV persistence or recurrence in some patients [23]. This hypothesis is also compatible with longitudinal observations that childbirth is associated with increased CIN3+ risk among women with persistent HPV infection [24]. Given the retrospective design, standardized colposcopy mapping variables (e.g., transformation zone type, quantified ectropion area, or cervical lacerations/scarring) were not available; these factors should be prospectively documented and tested in future studies. This finding should be interpreted cautiously but prompts further research, for instance, examining if postpartum timing or breastfeeding status influences PDT efficacy, or if cervical microenvironment differences in parous women play a role.

Collectively, the risk factor analysis from our study dovetails with prior evidence on what influences cervical disease outcomes and highlights important considerations for clinical practice. HPV genotype merits a brief discussion as well. While single-genotype infections had better outcomes than multi-genotype infections, we did not find that any specific high-risk type (HPV16 vs. HPV18) was significantly associated with treatment success in a multivariate sense. Earlier studies suggested HPV16 may be the most persistent and difficult to eradicate type [19]. Interestingly, in the context of PDT, some reports have shown no significant difference in clearance between HPV16 and other types when adequate follow-up is provided [13]. For example, in the LSIL PDT study from Shanghai, HPV16/18-positive cases had a slightly higher clearance at 3 months than others, and by 6 months, their clearance (94.9%) was statistically equivalent to non-16/18 cases (92.3%) [13]. Our high overall HPV clearance rate (91%) suggests that PDT, especially with a broad-acting photosensitizer and proper technique, can eliminate even the traditionally recalcitrant genotypes. This is a significant advantage, as HPV eradication is correlated with reduced recurrence and progression risk [16,17]. Indeed, the ability of PDT to *simultaneously* treat the lesion and clear underlying HPV infection is a distinguishing benefit over some surgical treatments (which remove diseased tissue but do not address field infection in adjacent epithelium). This “field clearance” effect of PDT may explain the low recurrence rates observed. Our Kaplan–Meier analysis showed that the vast majority of patients remained HPV-free and lesion-free over the follow-up period; the median time to recurrence was not reached in most subgroups, indicating durable responses. This is in line with other studies where long-term follow-up after PDT has shown sustained remission. In a 2-year follow-up study, Inada et al. found only 10% of treated CIN2/3 patients experienced any recurrence within two years, and 0% progressed to cancer, whereas 90% remained disease-free [19]. Even for CIN1, >75% had durable clearance at 2 years, with the remainder mostly showing only minor persistent lesions [19]. Our study’s median follow-up (~9.6 months, with some patients followed up to 8+ years) shows similarly encouraging durability, with only a small fraction of patients experiencing lesion persistence or reappearance. Furthermore, when comparing PDT to standard therapies, long-term efficacy appears comparable. Liu et al. reported no significant difference in 2-year cure and HPV eradication rates between PDT and LEEP for HSILs (both approximately 95% HPV clearance by 24 months) [17], but the PDT group avoided the complications inherent to excisional surgery. This underscores that PDT’s clinical benefits are not achieved at the cost of higher recurrence—on the contrary, PDT may reduce recurrences by sanitizing the tissue of HPV infection beyond the immediate lesion.

PDT was generally well tolerated in our cohort with no severe systemic complications. Pain management during PDT was individualized. While the procedure was routinely performed under local anesthesia, spinal anesthesia was offered and administered upon patient request to improve procedural comfort, allowing avoidance of general anesthesia. Although PDT is organ-preserving, late complications such as cervical stenosis may still occur, with an incidence in our cohort comparable to that reported after excisional procedures. Patients adhered to post-PDT photosensitivity precautions without incident, and only minimal transient side effects were noted, such as mild pelvic cramping or discharge consistent with epithelial sloughing. This matches reports from other centers: Qian et al. noted no serious adverse reactions in 40 HSIL patients treated with ALA-PDT, only slight transient discomfort in a few cases [12]. Randomized trials have similarly found PDT to be well-tolerated; Hillemanns et al. reported that HAL-PDT was “well accepted” by patients, with only self-limited local effects and no significant laboratory or systemic safety issues [18]. Compared to surgery, PDT avoids anesthesia risks and intraoperative or immediate postoperative complications. Our comparative observations (though non-randomized) echo those of Wang et al., who documented a “notably lower incidence of side effects” in PDT vs. conization [16]. The chief drawbacks of PDT noted were logistical in Wang’s study; PDT had a longer total treatment duration and higher cost than surgery [16]. In our setting, PDT was delivered in an outpatient context and, while the upfront cost of photosensitizer and laser time is considerable, it potentially offsets costs of surgical theater use and management of surgical complications. Nevertheless, economic analyses will be important in the future to justify the widespread adoption of PDT. From the patient’s perspective, the avoidance of fertility-threatening complications and the relatively atraumatic experience of PDT (no excisional pain, minimal downtime) are significant advantages that improve quality of life.

The clinical implications of our findings are significant. PDT can now be considered a valid therapeutic option for HPV-related cervical lesions, from low-grade abnormalities to high-grade precancerous lesions, especially in populations where fertility preservation or surgical risk avoidance is a priority. Young women with HSIL, in particular, form a key demographic who may benefit from PDT over conization [12,16]. The evidence from our study and others indicates that PDT offers comparable efficacy to excisional treatment in eradicating lesions and HPV [16,17], while conferring added benefits in terms of safety and cervical conservation. Thus, in a setting with the necessary expertise and equipment, PDT could be presented to patients as an alternative to LEEP/cone biopsy. It will be important for clinicians to stratify patients based on the risk factors discussed: for example, a patient with a single HPV16 infection, exophytic HSILs on the ectocervix, and no prior pregnancies appears to be an ideal PDT candidate with a high likelihood of cure. Conversely, a patient with multifocal lesions extending into the canal, infected by HPV16/18/52 simultaneously, and with a history of multiple pregnancies might have a relatively lower success probability with PDT alone; such a patient could still undergo PDT, but with counseling that repeat treatments or a backup excisional procedure might be needed if clearance is not achieved. Our data, coupled with the immunological findings from Ju et al., also hint at the possibility of combining PDT with other modalities to improve outcomes in tougher cases [15]. For instance, therapeutic HPV vaccines or immune checkpoint modulators might be used alongside PDT to boost the host response against HPV-infected cells. Since PDT can induce immunogenic cell death and enhance antigen presentation, it could synergize with immunotherapy, an exciting avenue for future research.

Looking forward, there are several directions for future work and improvements in the field of PDT for cervical disease. First, standardized, large-scale clinical trials are needed to further validate efficacy and optimize protocols. A randomized controlled trial comparing PDT vs. LEEP in a Western population would be valuable and could pave the way for international guidelines to incorporate PDT. Such trials should also evaluate long-term outcomes (5-year recurrence and perhaps even cancer incidence) to fully establish durability. Second, studies should focus on protocol optimization: determining the ideal photosensitizer, the optimal dosing and incubation time, and whether multiple treatment sessions yield better outcomes than a single session for high-grade lesions. For example, the difference between our approach (chlorin e6 intravenous PDT in one session) and others (fractionated ALA-PDT in multiple weekly sessions) needs to be delineated in terms of efficacy, patient compliance, and cost. Third, given the importance of treating endocervical disease, technology development is warranted, such as improved intra-cervical light delivery devices or endoscopic/light-diffusing fiber techniques to ensure the full transformation zone is treated even in TZ3 cases. Additionally, adjunctive mechanical removal of mucus or acetowhite mapping under colposcopy before PDT could help target occult lesions. Fourth, exploring combination therapy is a promising frontier: combining PDT with antiviral or immune-based therapies (imiquimod, interferon, therapeutic vaccines) might significantly improve clearance of stubborn infections and further reduce recurrence. There is also interest in photodynamic diagnosis using fluorescence to delineate lesions, which was part of our protocol to confirm photosensitizer uptake; this could be expanded to guide more selective treatment and spare normal areas. Lastly, cost-effectiveness and implementation research will be critical. Training programs for PDT, cost reduction in photosensitizers, and ensuring access in low-resource settings will determine how widely this therapy can be adopted. Interestingly, PDT is relatively low-cost in some analyses because it can be done in-office with minimal disposables; as technology matures, it may become an affordable option even in middle-income regions, complementing the WHO strategy to eliminate cervical cancer through not just vaccination and screening, but also effective treatment of pre-cancers.

### Limitations

This study has several limitations. First, it was a retrospective, single-center analysis, which carries an inherent risk of selection bias and external validity. The design may affect the generalizability of our findings to broader populations. Second, we did not include a randomized control group (such as a standard LEEP treatment arm), preventing direct comparison of PDT outcomes with the current standard of care. The absence of a concurrent control limits our ability to definitively attribute differences in outcomes to PDT alone, underscoring the need for future controlled trials. Third, the median follow-up in our cohort was relatively short (9.6 months), which may be insufficient to fully assess long-term recurrent rates. Although all patients included in the analysis were HIV-negative, smoking status, a well-known cofactor for HPV persistence and cervical neoplasia, could not be reliably assessed due to incomplete documentation in the medical records and was therefore not included in the analysis. This gap may have limited our ability to account for behavioral and immunological factors that influence treatment outcomes. Future prospective studies should systematically collect data on smoking and other immunosuppressive conditions to better refine risk stratification after PDT. Moreover, none of the patients included in the study received HPV vaccination around the time of PDT. Therefore, the observed HPV clearance can be attributed to PDT itself rather than vaccine-induced viral elimination [25]. We acknowledge that the lack of vaccinated individuals means our findings apply to an unvaccinated population; future studies might explore the interaction between PDT and vaccination status.

Finally, standardized colposcopy documentation of cervical anatomy (such as transformation zone type, extent of ectropion, or postpartum cervical changes) was not available due to the retrospective design. This prevented us from assessing how such anatomical variables might predict HPV clearance or disease recurrence after PDT. Future prospective studies should address these issues by incorporating a control arm for direct comparison, extending the duration of follow-up, standardizing follow-up schedules, and systematically collecting data on key cofactors (smoking, immunosuppressive conditions) as well as detailed cervical anatomy. Such measures would improve risk stratification and the overall interpretability of outcomes following PDT in cervical intraepithelial neoplasia.

## 5. Conclusions

Our study adds to the growing body of evidence that photodynamic therapy is a clinically efficacious and safe treatment for HPV-related cervical lesions. The outcomes we observed, high HPV clearance, high lesion regression, and low recurrence rates, mirror those of traditional surgical approaches [16,17] while offering the benefits of a tissue-sparing, minimally invasive procedure. The identification of certain risk factors (multiple HPV infection, endocervical involvement, etc.) provides insight into which patients might need tailored approaches or more intensive follow-up. Overall, PDT represents a valuable tool in the gynecologic oncology armamentarium, one that addresses an unmet need for fertility-preserving therapy. As research continues to refine this modality, we anticipate that PDT will move from an investigational therapy to an accepted standard option, fundamentally improving the management of HPV-associated cervical disease and contributing to the broader effort of cervical cancer prevention and elimination.

## Figures and Tables

**Table 1:** Univariate analysis of demographic characteristics, HPV status, and viral results after PDT.

Characteristics	Groups			*p* Value
	HPV Infection with ASCs	LSIL	HSIL	
Age, years				
M ± SD	31.4 ± 7.2	31.4 ± 8	34.9 ± 8.3	<0.001
Me [IQR]	31 [27–36]	31 [25–35]	34 [29–39]	<0.001
Age groups:				<0.001
18–25, *n* (%)	14 (18.7%)	33 (25.4%)	59 (9.7%)	
26–35, *n* (%)	42 (56%)	65 (50%)	307 (50.7%)	
36–45, *n* (%)	16 (21.3%)	25 (19.2%)	183 (30.2%)	
46–55, *n* (%)	3 (4%)	4 (3.1%)	43 (7.1%)	
56+, *n* (%)	0 (0%)	3 (2.3%)	14 (2.3%)	
Age at sexual debut, years				
M ± SD	19 ± 3.4	18.5 ± 2.7	18.1 ± 2.3	0.047
Me [IQR]	18 [17–20]	18 [17–19]	18 [17–19]	0.21
Age at sexual debut groups:				0.24
<18, *n* (%)	22 (30.1%)	51 (41.1%)	233 (40.0%)	
≥18, *n* (%)	51 (69.9%)	73 (58.9%)	350 (60.0%)	
Number of sexual partners				
M ± SD	4 ± 4	4 ± 3	5 ± 5	0.002
Me [IQR]	3 [1–5]	3 [2–5]	4 [2–7]	0.011
Sexual partners:				0.13
1, *n* (%)	19 (26.4%)	24 (19.5%)	87 (15.1%)	
2–3, *n* (%)	19 (26.4%)	38 (30.9%)	169 (29.3%)	
>3, *n* (%)	34 (47.2%)	61 (49.6%)	321 (55.6%)	
Pregnancy status, *n* (%)	42 (58.3%)	72 (57.6%)	431 (72.9%)	<0.001
No. of pregnancies, Me [IQR]	1 [0–2]	1 [0–2]	1 [0–3]	<0.001
Pregnancies:				0.15
1, *n* (%)	48 (66.7%)	79 (63.2%)	323 (54.7%)	
2–3, *n* (%)	18 (25.0%)	34 (27.2%)	185 (31.3%)	
>3, *n* (%)	6 (8.3%)	12 (9.6%)	83 (14.0%)	
TZ types:				0.84
TZ1, *n* (%)	22 (29.3%)	39 (31.0%)	186 (31.6%)	
TZ2, *n* (%)	20 (26.7%)	37 (29.4%)	183 (31.1%)	
TZ3, *n* (%)	33 (44.0%)	50 (39.7%)	220 (37.4%)	
Main HPV genotype:				<0.001
*HPV16*, *n* (%)	25 (50.0%)	46 (47.9%)	297 (67.7%)	
*HPV18*, *n* (%)	4 (8.0%)	5 (5.2%)	18 (4.1%)	
*HPV31*, *n* (%)	3 (6.0%)	7 (7.3%)	34 (7.7%)	
*HPV33*, *n* (%)	2 (4.0%)	6 (6.2%)	29 (6.6%)	
Other genotypes, *n* (%)	16 (32.0%)	32 (33.3%)	61 (13.9%)	
HPV infection:				<0.001 ^a^
Single, *n* (%)	33 (44.0%)	54 (41.5%)	310 (51.2%)	
Multiple, *n* (%)	24 (32.0%)	54 (41.5%)	185 (30.5%)	
Not detected, *n* (%)	14 (18.7%)	14 (10.8%)	34 (5.6%)	
No data, *n* (%)	4 (5.3%)	8 (6.2%)	77 (12.7%)	
Multiple HPV infection:				0.86
2–3, *n* (%)	14 (87.5%)	33 (82.5%)	104 (81.9%)	
>3, *n* (%)	2 (12.5%)	7 (17.5%)	23 (18.1%)	
Viral load				
M ± SD	27.8 ± 133.3	13.9 ± 45.5	15.8 ± 62.6	0.59
Me [IQR]	5.4 [4.5–6.1]	6.1 [5.4–7.1]	6 [5.1–7]	0.038

ASCs—atypical squamous cells; HPV—human papillomavirus; HSIL—high-grade squamous intraepithelial lesion; IQR—interquartile range; LSIL—low-grade squamous intraepithelial lesion; M—mean; Me—median; NILM—negative for intraepithelial lesion and malignancy; SD—standard deviation; TZ—transformation zone; ^a^ comparison excludes data with unknown HPV testing results.

**Table 2:** Characteristics of logistic regression models of complete response, HPV clearance and lesion remission.

LR Models	Pseudo-R^2^	ROC-AUC	95% CI	*p* Value	Se, %	Sp, %
Complete response	0.104	0.69	0.62–0.75	<0.001	61	62.8
HPV clearance	0.064	0.62	0.54–0.69	<0.001	60.3	55.4
Lesion remission	0.029	0.60	0.50–0.70	0.006	78.2	40.5

CI—confidential interval; ROC-AUC—receiver operating characteristic area under curve; HPV—human papillomavirus; Se—sensitivity; Sp—specificity.

**Table 3:** Characteristics of the relationship between predictors and the odds of CR.

Predictors	Unadjusted		Adjusted	
	COR; 95% CI	*p* Value	AOR; 95% CI	*p* Value
Age groups:				
26–35	1.73; 0.92–3.25	0.088	2.18; 1.07–4.44	0.032
36–45	1.43; 0.73–2.82	0.302	1.60; 0.69–3.69	0.27
46–55	2.07; 0.65–6.60	0.217	2.64; 0.73–9.55	0.138
56+	1.28; 0.26–6.25	0.757	1.40; 0.26–7.44	0.697
Pregnancy status	0.70; 0.42–1.18	0.179	0.46; 0.25–0.86	0.014
TZ types:				
TZ2	1.80; 0.99–3.26	0.054	1.98; 1.06–3.69	0.033
TZ3	1.22; 0.73–2.04	0.443	1.12; 0.64–1.97	0.686
SILs grade:				
LSIL	1.96; 0.90–4.26	0.09	2.47; 1.10–5.54	0.029
HSIL	2.66; 1.43–4.96	0.002	3.18; 1.65–6.12	0.001
Multiple HPV infection:				
2–3 genotypes	0.53; 0.31–0.89	0.017	0.44; 0.25–0.77	0.004
>3 genotypes	0.19; 0.09–0.44	<0.001	0.16; 0.07–0.38	<0.001

ASCs—atypical squamous cells; AOR—adjusted odds ratio; CI—confidential interval; COR—crude odds ratio; HPV—human papillomavirus; HSIL—high-grade squamous intraepithelial lesion; LSIL—low-grade squamous intraepithelial lesion; SILs—squamous intraepithelial lesions; TZ—transformation zone. Reference category: age group—18–25; TZ type—TZ1; SILs grade—ASCs with HPV infection; single HPV infection vs. multiple HPV infections.

**Table 4:** Characteristics of the relationship between predictors and the odds of HPV clearance.

Predictors	Unadjusted		Adjusted	
	COR; 95% CI	*p* Value	AOR; 95% CI	*p* Value
SILs grade:				
LSIL	1.94; 0.83–4.54	0.125	2.13; 0.90–5.06	0.087
HSIL	2.90; 1.47–5.74	0.002	3.07; 1.53–6.13	0.002
Multiple HPV infection:				
2–3 genotypes	0.90; 0.46–1.75	0.755	0.93; 0.47–1.82	0.821
>3 genotypes	0.18; 0.08–0.43	<0.001	0.18; 0.08–0.42	<0.001

ASCs—atypical squamous cells; AOR—adjusted odds ratio; CI—confidential interval; COR—crude odds ratio; HPV—human papillomavirus; HSIL—high-grade squamous intraepithelial lesion; LSIL—low-grade squamous intraepithelial lesion; SILs—squamous intraepithelial lesions. Reference category: SILs grade—ASCs with HPV infection; single HPV infection vs. multiple HPV infections.

**Table 5:** Characteristics of the relationship between predictors and the odds of lesion remission.

Predictors	Unadjusted		Adjusted	
	COR; 95% CI	*p* Value	AOR; 95% CI	*p* Value
Multiple HPV infection:				
2–3 genotypes	0.47; 0.22–0.99	0.047	0.47; 0.22–0.99	0.047
>3 genotypes	0.25; 0.08–0.78	0.017	0.25; 0.08–0.78	0.017

AOR—adjusted odds ratio; CI—confidential interval; COR—crude odds ratio; HPV—human papillomavirus. Reference category: single HPV infection vs. multiple HPV infections.

**Table 6:** Hazard ratio of partial response to independent predictors.

Predictors	Unadjusted		Adjusted	
	HR; 95% CI	*p* Value	HR; 95% CI	*p* Value
Age groups:				
26–35	0.55; 0.31–0.99	0.047	0.37; 0.19–0.70	0.002
36–45	0.58; 0.31–1.09	0.09	0.41; 0.20–0.87	0.02
46–55	0.43; 0.14–1.29	0.131	0.30; 0.09–0.98	0.046
56+	0.92; 0.21–4.01	0.913	0.80; 0.16–3.70	0.78
Pregnancy status	1.36; 0.83–2.22	0.225	2.23; 1.26–3.95	0.006
TZ types:				
TZ2	0.57; 0.33–1.01	0.052	0.55; 0.31–0.97	0.04
TZ3	0.84; 0.52–1.36	0.477	0.95; 0.56–1.59	0.837
SILs grade:				
LSIL	0.55; 0.27–1.12	0.099	0.43; 0.21–0.89	0.023
HSIL	0.39; 0.22–0.68	<0.001	0.34; 0.19–0.62	<0.001
Multiple HPV infection:				
2–3 genotypes	2.12; 1.29–3.47	0.003	2.36; 1.42–3.93	<0.001
>3 genotypes	3.95; 2.01–7.76	<0.001	4.70; 2.33–9.48	<0.001

CI—confidence interval; HPV—human papillomavirus; HR—hazard ratio; HSIL—high-grade squamous intraepithelial lesion; LSIL—low-grade squamous intraepithelial lesion; SILs—squamous intraepithelial lesions; TZ—transformation zone. Reference category: age group—18–25; TZ type—TZ1; SILs grade—ASCs with HPV infection; single HPV infection vs. multiple HPV infections.

**Table 7:** Hazard ratio of persistent HPV infection on independent predictors.

Predictors	Unadjusted		Adjusted	
	HR; 95% CI	*p* Value	HR; 95% CI	*p* Value
Age groups:				
26–35	0.57; 0.29–1.12	0.102	0.43; 0.21–0.90	0.025
36–45	0.48; 0.23–1.02	0.057	0.37; 0.16–0.89	0.025
46–55	0.56; 0.18–1.75	0.319	0.47; 0.14–1.57	0.22
56+	1.25; 0.28–5.58	0.773	1.15; 0.24–5.48	0.857
Pregnancy status	1.22; 0.70–2.13	0.481	1.96; 1.03–3.72	0.04
SILs grade:				
LSIL	0.55; 0.25–1.20	0.13	0.42; 0.19–0.94	0.036
HSIL	0.35; 0.19–0.65	<0.001	0.30; 0.16–0.58	<0.001
Multiple HPV infection:				
2–3 genotypes	1.35; 0.71–2.57	0.362	1.32; 0.68–2.54	0.411
>3 genotypes	4.30; 2.10–8.83	<0.001	5.01; 2.41–10.42	<0.001

CI—confidence interval; HPV—human papillomavirus; HR—hazard ratio; HSIL—high-grade squamous intraepithelial lesion; LSIL—low-grade squamous intraepithelial lesion; SILs—squamous intraepithelial lesions; TZ—transformation zone. Reference category: age group—18–25; SILs grade—ASCs with HPV infection; single HPV infection vs. multiple HPV infections.

**Table 8:** Hazard ratio of lesion remission on independent predictors.

Predictors	Unadjusted		Adjusted	
	HR; 95% CI	*p* Value	HR; 95% CI	*p* Value
Age groups:				
26–35	0.48; 0.20–1.12	0.088	0.32; 0.13–0.83	0.018
36–45	0.59; 0.24–1.43	0.241	0.41; 0.15–1.14	0.088
56+	0.92; 0.11–7.32	0.933	0.72; 0.09–6.20	0.768
Pregnancy status	1.40; 0.66–2.97	0.378	2.40; 1.01–5.70	0.048
Multiple HPV infections:				
2–3 genotypes	2.38; 1.15–4.93	0.02	2.61; 1.23–5.52	0.012
>3 genotypes	3.76; 1.29–10.92	0.015	4.21; 1.44–12.31	0.009

CI—confidence interval; HPV—human papillomavirus; HR—hazard ratio; HSIL—high-grade squamous intraepithelial lesion; LSIL—low-grade squamous intraepithelial lesion; SILs—squamous intraepithelial lesions; TZ—transformation zone. Reference category: age group—18–25; single HPV infection vs. multiple HPV infections.

## Data Availability

The raw data supporting the conclusions of this article will be made available by the authors on request.

## Abbreviations

The following abbreviations are used in this manuscript:

ASC	Atypical squamous cell
CI	Confidence interval
CR	Complete response
HPV	Human papillomavirus
HR	Hazard ratio
HSIL	High-grade squamous intraepithelial lesion
LSIL	Low-grade squamous intraepithelial lesion
NILM	Negative for intraepithelial lesions or malignancies
OR	Odds ratio
PDT	Photodynamic therapy
SIL	Squamous intraepithelial lesion
TZ	Transformation zone
